# Gastric Cancer and the Daily Intake of the Major Dish Groups Contributing to Sodium Intake: A Case-Control Study in Korea

**DOI:** 10.3390/nu13041365

**Published:** 2021-04-19

**Authors:** Jung-Hyun Kwak, Chang-Soo Eun, Dong-Soo Han, Yong-Sung Kim, Kyu-Sang Song, Bo-Youl Choi, Hyun-Ja Kim

**Affiliations:** 1 Department of Food and Nutrition, Gangneung-Wonju National University College of Life Science, 7 Jukheon-gil, Gangneung-si, Gangwon-do 25457, Korea; hyun4615@hanmail.net; 2 Department of Internal Medicine, Hanyang University College of Medicine, 222 Wangsimni-ro, Seongdong-gu, Seoul 04763, Korea; cseun@hanyang.ac.kr (C.-S.E.); hands@hanyang.ac.kr (D.-S.H.); 3 Division of Gastroenterology, Department of Internal Medicine, Hanyang University Guri Hospital, 153 Gyeongchun-ro, Guri-si, Gyeonggi-do 11923, Korea; 4 Funtional Genomics Institute, PDXen Biosystems Co., ETRI Convergence Commercialization Center, 218 Gajeong-ro, Yuseong-gu, Daejeon 34129, Korea; yskakim@gmail.com; 5 Department of Pathology, Chungnam National University College of Medicine, 99 Daehak-ro, Yuseong-gu, Daejeon 34134, Korea; qsong@cnu.ac.kr; 6 Department of Preventive Medicine, Hanyang University College of Medicine, 222 Wangsimni-ro, Seongdong-gu, Seoul 04763, Korea

**Keywords:** gastric cancer, high-sodium dish groups, case-control study, sodium intake, smoking, alcohol drinking

## Abstract

Studies on the association between gastric cancer (GC) and the intake of soup-based dish groups (noodles and dumplings, soups, and stews), which are sodium-contributing foods, in Korea are insufficient, and the results of studies on the intake of pickled vegetables such as kimchi are inconsistent. This study aimed to determine the association between the incidence of GC and the daily intake of high-sodium dish groups (noodles and dumplings, soups, stews, and pickled vegetables) and whether these associations differ depending on behavioral risk factors for GC. In this case-control study, subjects aged 20–79 years were recruited from two hospitals between December 2002 and September 2006. A total of 440 cases and 485 controls were recruited, of which 307 pairs were matched and included for the analysis. In our results, a higher intake of noodles and dumplings was associated with a significantly increased incidence of GC. In the participants who consumed past or current alcohol, a higher intake of noodles and dumplings was associated with a significantly increased incidence of GC. Our results suggest that efforts to reduce the daily sodium intake from noodles and dumplings are needed to prevent and reduce the incidence of GC.

## 1. Introduction

Based on GLOBOCAN 2020 data, gastric cancer (GC) is the fifth most common cancer and fourth for mortality globally, with an estimated 769,000 deaths in 2020 [1]. In particular, Korea has the highest incidence of GC [2]. According to Statistics Korea, the estimated crude incidence of gastric cancer (GC) was 57.1/100,000 persons in 2018, which was still higher than that of other cancers such as lung and liver cancer [3].

Recently, a systematic review reported the following as risk factors for GC: diet, lifestyle, genetic predisposition, family history, treatment and medical conditions, and *Helicobacter pylori* (*H. pylori*) infections [4]. The World Cancer Research Fund International (2018) reported that a higher consumption of foods preserved by salting may be a cause of GC [5]. In addition, several studies have reported that a high intake of salty foods or salts increases the risk of GC [6,7,8,9,10], and experimental studies support these findings [6,11]. On the other hand, our previous study and others found no association between salty food [12] or salt [13,14] and GC. Some salty foods, such as fermented foods, contain antioxidant nutrients that are beneficial to health [15], and each study categorized salty foods slightly differently, which may have contributed to the inconsistency in the results [7,8,10,12]. In addition, it is difficult to measure an individual’s exact sodium intake due to inaccuracies during measurement using dietary questionnaires.

According to Korea Health Statistics 2019, the daily intake of sodium is 3512.3 mg (aged ≥ 19 years) [16], which is more than twice the adequate intake (1500 mg) as set in the 2020 Dietary Reference Intakes for Koreans [17]. The Korea National Health and Nutrition Examination Survey (KNHANES) VI (2013–2015) (aged ≥ 6 years) presented noodles and dumplings, pickled vegetables, soups, and stews from 1st to 4th as major dish groups that contribute to sodium intake [18]. These data suggest that soup-based dish groups, such as noodles, soups, and stews, greatly contribute to sodium intake in Korea. The higher consumption of sodium by Koreans than inhabitants of other countries may be due to their soup-based diet as well as traditional pickled foods [19,20]. However, studies on the association between GC and soup-based dish groups are insufficient [21], and the results of studies on pickled vegetables, such as kimchi, are inconsistent [12,22,23].

In addition, smoking and alcohol consumption are well-known behavioral risk factors for GC [5]. Additionally, these behavioral factors are associated with unhealthy dietary patterns [24]. Some studies have reported that smokers or alcohol drinkers prefer salty food [25], and they have a low intake of fruits and dairy products and a high intake of fast food [26]. Smoking and alcohol consumption can act as carcinogenic factors [27], but when they are combined with unhealthy dietary habits, their effect on GC incidence may be worse.

Therefore, the primary goal of the present study was to determine the association between the incidence of GC and the daily intake in major dish groups that contribute to sodium intake. The secondary goal of this study was to evaluate whether the behavioral risk factors of GC (i.e., smoking and alcohol consumption) influence the association between the incidence of GC and the daily intake of major dish groups that contribute to sodium intake.

## 2. Materials and Methods

### 2.1. Study Population

Subjects aged 20–79 years were recruited from the Chungnam University Hospital and Hanyang University Guri Hospital between December 2002 and September 2006. Due to the slight change in the questionnaire items during the study period, the subjects were recruited in the following stages: 1st (December 2002 to August 2003) and 2nd (October 2003 and September 2006) stages. GC was histologically diagnosed based on the World Health Organization classification of tumors of the digestive system, as follows [28]: a gastroscopy was performed by a gastroenterologist and the final diagnosis of GC was confirmed by a pathologist through a biopsy. The control group comprised those who visited the same hospital during the same period and did not have any gastric problems. A total of 440 cases and 485 controls were recruited, of which 316 pairs were matched at a ratio of 1:1 by hospital, age within 5 years, sex, and study admission period within 1 year. Among the 316 pairs, the pairs with daily energy intakes of < 500 kcal (five cases) or > 5000 kcal (one case and two controls) and those with missing information on the intake of noodles and dumplings (one case) were excluded. Finally, 307 pairs (124 pairs for Chungnam University Hospital; 183 pairs for Hanyang University Guri Hospital) were included for this analysis. All participants provided written informed consent, and the study protocol was approved by the Institutional Review Board of Hanyang University Medical Center (IRB no. 2003-4).

### 2.2. Data Collection

The questionnaire included questions on sociodemographic characteristics (i.e., sex, age, education level, etc.), behavioral factors (i.e., smoking and alcohol consumption), disease status, family history, and dietary habits. Height and weight were collected from the medical records and questionnaires. Body mass index (BMI) was computed as weight in kilograms divided by height in meters squared (kg/m^2^). We grouped the participants into four based on their BMI: “obese”, ≥25 kg/m^2^; “overweight”, 23.0–24.99 kg/m^2^; “normal weight”, 18.5–22.99 kg/m^2^; and “underweight”, ≤18.49 kg/m^2^. *H. pylori* infection was tested with the Campylobacter-like organism (CLO) test kit (Product no: 60480; Kimberly-Clark/Ballard Medical Products, Draper, UT, USA), a rapid urea degradation test. This test was performed when tissue collection was possible.

### 2.3. Dietary Data

The data on dietary factors were collected using a quantitative food frequency questionnaire (FFQ). The FFQs used for the 1st and 2nd stages had slightly different survey items and methods. For the first stage, the FFQ assessed 103 food or dish items, with an intake period (1–12 months) of 1 year, frequency of food consumption per month, week, or day, and average serving size. For the second stage, the FFQ comprised 116 food or dish items, frequency of food consumption with nine categories (“never or less than once a month”, “1–3 times a month”, “1 time a week”, “2–4 times a week”, “5–6 times a week”, “once a day”, “2–3 times a day”, “4–5 times a day”, and “≥6 times a day”), and average serving size. Therefore, we extracted the common food items for the first stage based on the second stage. After that, the food intake or nutrient intake was calculated for each of the stages, divided into three quartiles, and merged. This was converted into daily intake by considering the intake period and frequency, and the intake of each of the investigated foods. All questionnaires were administered by a well-trained interviewer and investigated the frequency and amount of food consumed during 12 months of 3 years before the date of the interview to assess remote dietary intake. The total energy intake for each food was estimated using the Korean Foods and Nutrients Database [29]. The dish groups with high sodium content were used as the four major dish groups (noodles and dumplings, pickled vegetables, soups, and stews) that contribute to the sodium intake of Koreans based on the 6th KNHANES data as follows [18]. First, noodles and dumplings were part of eight dishes, as follows: 1. Noodles, banquet noodles (janchi-guksu), cold noodles (naengmyeon), and noodles in cold soybean soup (kong-guksu); 2. spicy mixed noodles, spicy noodles (bibim-guksu), spicy cold chewy noodles (jjolmyeon), and cold noodles with spicy dressing (bibim-naengmyeon); 3. instant noodles (ramen); 4. noodles with black bean sauce (jajangmyeon); 5. Chinese-style noodles with vegetables and seafood (jjamppong); 6. hand-pulled dough soup (sujebi) and chopped noodles (kal-guksu); 7. dumplings and dumpling soups; and 8. sliced rice cake soup (tteok-guk). Second, pickled vegetables comprised seven dishes, as follows: 1. Chinese cabbage kimchi (baechu-kimchi); 2. radish kimchi; seasoned cubed radish roots (kkakduki), young summer radish kimchi (yeolmu-kimchi); 3. white kimchi (baek kimchi); 4. pickled radish (dongchimi); 5. sesame leaf kimchi; 6. cucumber kimchi; and 7. pickles (cucumber, red pepper, garlic, radish, etc.). Third, soups comprised nine dishes, as follows: 1. beef soup; 2. beef bone soup (gomguk), ox bone soup (seolleong-tang), and short rib soup (galbi-tang); 3 spicy beef soup (yukgaejang) and hangover soup (haejangguk); 4. chicken soup with ginseng (samgye-tang) and braised spicy chicken (dak-dori-tang); 5. vegetable soybean paste soup; 6. bean sprouts soup (kongnamul-guk) and bean sprout kimchi soup (kongnamul kimchi-guk); 7. cold wakame soup (miyeok naengguk) and seaweed soup (miyeok-guk); 8. dried cod soup (bugeoguk); and 9. invigorating soup (boshintang). Fourth, stews comprised four dishes, as follows: 1. kimchi stew (kimchi jjigae); 2. soybean paste stew (doenjang jjigae) and fermented soybean paste stew (cheonggukjang jjigae); 3. pollack stew; and 4. other fish stews (mackerel, saury, blacktail, etc.). The daily intake of each dish was calculated by multiplying the frequency per day and intake amount and summing them to estimate the total daily intake of dish groups.

The dietary questionnaire included items on salty taste preference, with a 5-point Likert scale: (1) not favored at all; (2) slightly not favored; (3) no opinion; (4) slightly favored; and (5) strongly favored. The five responses on the salty taste preferences were classified into three categories: (1) not favored at all and slightly not favored; (2) no opinion; and (3) slightly favored and strongly favored.

### 2.4. Statistical Analysis

The statistical analysis was performed using the SAS 9.4 version (SAS Institute Inc., Cary, NC, USA). To compare the general characteristics of the cases and controls, the Student’s *t*-test was used to analyze the continuous variables and the Chi-squared test was used for categorical variables. The continuous and categorical variables are presented as mean values ± standard deviation and numbers (percentages), respectively. Since there was a slight difference between the FFQs for the first and second stages, the tertiles of each dish group were applied according to each stage. The odds ratios (OR) and 95% confidence intervals (CI) for the GC risk were calculated across the tertiles for each high-sodium dish group using logistic regression after controlling for known risk factors. The lowest tertile of each high-sodium dish group was used as a reference. To test for trends, the median values of each tertile category of the high-sodium dish groups were used as continuous variables. We analyzed the OR for GC according to the salty food preference of the participants who answered “no opinion” as the reference group. Model I was adjusted for age (as a continuous variable) and sex. Model II was further adjusted for BMI (≤18.49, 18.5–22.99, 23.0–24.99, or ≥25), education level (≤elementary school, middle school, ≥ high school, or missing), family history (first-degree relatives) of GC (no, yes, or missing), smoking status (never, past, or current smoker), alcohol consumption (never, past, or current drinkers), total energy intake (as a continuous variable), and *H. pylori* infection (no, yes, or missing). In addition, we conducted a stratified analysis according to the GC risk factors (alcohol consumption or smoking status) for the fully adjusted model. During the stratification analysis, the stratified variables were excluded from the model. The current and past smokers were combined into the “ever smoked” group before analysis. Moreover, the current and past drinkers were combined into the “ever drank” group before analysis.

## 3. Results

### 3.1. General Characteristics of Cases and Controls

Table 1 describes the general characteristics of the 307 GC cases and 307 controls. The mean ages of the case and control groups were 56.9 (±12.0) years and 56.2 (±11.9) years, respectively. No difference was observed between the two groups based on sex and hospital visited due to the matching selection. In addition, no difference was observed between the cases and controls based on alcohol consumption, smoking status, education levels, and family history of GC. The case group had a lower prevalence of obesity (22.2%) than the control group (32.6%) (*p* = 0.014). The control group had a higher proportion of participants with *H. pylori* infection (45.6%) than the case group (31.6%) (*p* < 0.001). The case group consumed significantly more total energy than the control group (*p* = 0.002).

### 3.2. Association between Behavioral GC Risk Factors and GC

Table 2 shows the association between behavioral GC risk factors and the incidence of GC. Alcohol consumption was not significantly associated with the incidence of GC based on models I and II (model I: OR = 1.00, 95% CI = 0.67–1.49, model II: OR = 2.09, 95% CI = 0.76–5.73). Regarding smoking status, model I showed that the participants who had ever smoked had a significantly higher incidence of GC than those who had never smoked (OR = 1.73, 95% CI = 1.06–2.84). However, model II did not show that this was statistically significant (OR = 1.51, 95% CI = 0.62–3.66).

### 3.3. Association between High-Sodium Dish Groups and GC

Table 3 shows the association between the daily intake of high-sodium diet groups and the incidence of GC. For model I, the highest tertile of intakes of noodles and dumplings showed a significantly higher incidence of GC than the lowest tertile (OR = 1.98, 95% CI = 1.31–3.00, *p* for trend = 0.0005). For model II, the fully adjusted model, the highest tertile of intakes of noodles and dumplings showed a significantly higher incidence of GC than the lowest tertile (OR = 1.65, 95% CI = 1.03–2.63, *p* for trend = 0.017). The daily intakes of soups and stews showed no significant differences related to the incidence of GC. In contrast, medium (the 2nd tertile) intake of pickled vegetables was associated with a tendency of a decrease in the incidence of GC (OR = 0.67, 95% CI = 0.44–1.02, *p* for trend = 0.288).

### 3.4. Association between the Daily Intake of Noodles and Dumplings and GC Stratified by Behavioral GC Risk Factors

Table 4 shows the association between the daily intake of noodles and dumplings and the incidence of GC stratified by behavioral GC risk factors. When stratified by alcohol drinking status, the highest tertile of intakes of noodles and dumplings showed a significantly higher incidence of GC than the lowest tertile among participants who were past or current drinkers in the fully adjusted model (OR = 2.00, 95% CI = 1.12–3.56, *p* for trend = 0.017), but this association was not significant among those who had never drunk. When stratified by smoking status, the highest tertile of the intakes of noodles and dumplings showed a tendency of an increase in the incidence of GC compared with the lowest tertile among the participants with past or current smokers in the fully adjusted model (OR = 1.68, 95% CI = 0.92–3.05, *p* for trend = 0.083). This association was not significant in the participants who had never smoked.

### 3.5. Association between the Preference of Salty Food and GC

Table 5 shows the association between the preference for salty food and the incidence of GC. In model I, the participants who considered their salty food preference “slightly favored” and “strongly favored” showed a significantly increased incidence of GC than those had no opinion (OR = 1.54, 95% CI = 1.01–2.33, *p* for trend = 0.046). However, statistical significance was weakened in model II (OR = 1.51, 95% CI = 0.97–2.36, *p* for trend = 0.153).

## 4. Discussion

In our study, a higher daily intake of noodles and dumplings was associated with a higher incidence of GC. When stratified by alcohol consumption in participants who were past or current alcohol drinkers, a higher daily intake of noodles and dumplings was associated with a significantly increased incidence of GC.

Sodium is an essential element for homeostasis and physiological functioning in the body, but high sodium intake has been reported to cause health problems such as obesity [30], high blood pressure [31], cardiovascular disease [32], and GC [9]. Sodium intake is higher when added during cooking or consumed as part of dishes [33]. In addition, salt is not a direct carcinogen, but salty food, including food-derived carcinogens, can increase the incidence of GC [34]. Therefore, it is important to focus on dish groups that contribute to salt intake rather than the salt.

The higher consumption of sodium by Koreans than by inhabitants of other countries may be attributed to the high intake of traditional pickled foods and soup-based diets [19,20]. The KNHANES VI data and the outcomes of a study by Song et al. (2013) suggest that soup-based dish groups, such as noodles and dumplings, soups, and stews, contribute the most to sodium intake in Korea [18,20]. However, studies on major dish groups that contribute to sodium intake are rare.

Among the major dish groups with high sodium content, noodles and dumplings are among the most popular foods for Korean adults [35]. In a study on noodle consumption and health problems, a positive association was found between noodle consumption and non-alcoholic fatty liver disease [36], atopic dermatitis [37], cardiovascular disease [38], and GC [39]. In our study, a higher intake of noodles and dumplings increased the incidence of GC. A previous study examined the association between GC and the intake of noodles and bread [39]. In this Chinese case-control study, no association was found between the intake of noodles and bread and the risk of stomach cancer in either men (OR = 1.1, 95% CI = 0.9–1.5) or women (OR = 1.2, 95% CI = 0.8–1.8). However, unlike in our study that included noodles and dumplings in one category, they analyzed noodles and bread as part of one category. Therefore, there is a limit to comparing the results of our study with those of previous studies due to the differences in the dish groups. However, several mechanisms may account for the association between the daily intake of noodles and dumplings and the incidence of GC. First, this may be due to the high sodium content in noodles and dumplings. The KNHANES VI (2013–2015) data reported that noodles and dumplings (587 mg/day) contributed the most among the dish groups contributing to sodium intake [18]. Farrand et al. (2017) evaluated the sodium content of instant noodles by countries and reported that instant noodles contained too much sodium, especially in China, as the sodium content was 1944 mg/100 g [40]. In Korea, the sodium content of one serving size was 1606 mg/120 g of instant noodles, 956 mg/210 g of buckwheat noodles, 830 mg/210 g of noodles, and 549 mg/17 g of black bean sauce, so the sodium content of the sauce of noodles or the noodles is high [17]. In 2020, the Dietary Reference Intakes for Koreans suggests the adequate intake of sodium (aged 9–64 years) is 1500 mg/day, and the chronic disease risk reduction intake of sodium (aged 9–64 years) is 2300 mg/day [17]. However, the average sodium intake of Koreans was 3512.3 mg/day (aged ≥ 19 years) [16], which is higher than each standard. Several experimental studies [6,11] have shown that high sodium increases the incidence of GC as it (1) increases the colonization of gastric mucosa by *H. pylori* infection, (2) damages the mucous cell layer surface, (3) increases the expression of inflammatory mediators such as cyclooxygenase-2 and inducible nitric oxide, and (4) upregulates *cagA* expression, which leads to inflammation, hypochlorhydria, and enhanced carcinogenesis [11]. Second, this may also be due to the high starch content, unlike other salty foods such as kimchi, soups, and stews. Highly starchy food causes hyperglycemia and hyperinsulinemia, which increases oxidative stress and inflammatory responses [41], and can also lead to gastric carcinogenesis by upregulating glycolysis-related genes (*enolase 1*) [42]. Ye et al. (2017) reported that in the subgroup analysis, higher carbohydrate intake was significantly associated with increased GC risk in Asian countries [43]. Third, among noodles, instant noodles (ramen) are among the most frequently eaten foods by Koreans, but they have no vegetable content. Koreans usually boil soups and stews with several vegetables (onions, gallic, cabbages, pumpkins, bean sprouts, etc.) and fermented sauces such as soy sauces, soybean paste, or red chili paste. Since vegetables are a good source of various antioxidant nutrients, it is well-known that the low consumption of vegetables is associated with an increase in the incidence of GC [44,45]. In addition, although the fermented sauces contain a lot of sodium, they also contain nutrients that have anti-cancer effects [15].

The results of previous studies on pickled vegetables, such as kimchi, are inconsistent [12,22,23]. In some previous studies, kimchi had a protective effect against GC [12]. These protective effects may be attributed to the anticancer components of kimchi, such as lactic acid bacteria, fiber, and organosulfur compounds [46]. Meanwhile, in some meta-analyses, pickled vegetables were associated with an increased incidence of GC [22,23]. Pickled vegetables have high amounts of sodium as well as the aforementioned healthy nutrients, which may be associated with an increased incidence of GC. In our study, the moderate intake of pickled vegetables showed a tendency to decrease the risk of GC. This result suggests that an adequate amount of kimchi, rather than excessive intake, may help with health problems related to GC. However, this should be further clarified through further research.

In our study, there was no association between the incidence of GC and daily intake of soups and stews. Only one study reported that high-stewed foods, such as soybean paste stew and hot pepper-soybean stew, may increase the risk of GC, although they did not report for the daily intake of stewed foods [21]. In our study, the items included in soups and stews are different from those of previous studies, but we presented daily intakes of soups and stews and included the items of soup and stew that Koreans consume frequently. The effects of soups and stews on GC risk may differ depending on the vegetables in soups and stews, the content of fermented sauce, such as soybean paste, and the actual intake of a person.

Smoking and alcohol consumption are well-known behavioral risk factors for GC [3]. Additionally, these behavioral factors are associated with unhealthy dietary patterns [24]. Lampuré et al. (2015) reported that smokers or alcohol drinkers prefer salty food [20], and smokers have a low intake of fruits and dairy products and a high intake of fast food [21]. Smoking and alcohol consumption can act as carcinogenic factors [27], but when they are combined with unhealthy dietary habits, it is thought that their effect on GC incidence may be worse. Especially, one of the mechanisms of alcohol consumption that affects GC is the production of inflammatory markers (i.e., prostaglandins and lipid peroxidation) and the generation of oxidative stress substances such as oxygen radical species [2]. Since this mechanism is a reaction that increases in the body even with the intake of high starchy foods [41], it may have a synergistic effect on the incidence of GC in a subject with two factors such as increased noodle consumption and alcohol consumption. In the present study, when smoking or alcohol drinking status was analyzed without stratification, statistical significance could not be confirmed in the participants who had ever smoked and drunk alcohol after adjusting for confounding factors. However, when stratified by alcohol drinking or smoking status, we found that a higher intake of noodles and dumplings significantly increased the risk of GC (OR = 2.00, 95% CI = 1.12–3.56) in participants who had ever drunk alcohol and showed a tendency of increased risk of GC (OR = 1.68, 95% CI = 0.92–3.05) in participants who had ever smoked. From these results, we could confirm that the association between the intake of noodles and dumplings and GC increased in participants who had ever drunk alcohol or smoked. In addition, when stratified by family history of GC or *H. pylori* infection, we found that a higher intake of noodles and dumplings showed a tendency of increased risk of GC in participants with a family history of GC or with positive *H. pylori* infection (Table S1). Therefore, efforts to reduce excessive intake of noodles and dumplings in participants with GC risk factors are considered necessary.

In addition, several studies have reported an association between salty food preference and GC [7,47]. Kim et al. (2009) reported that the preference for a salty taste is related to the intake of salty food and its frequency [48]. We also confirmed that the risk of GC showed an increased tendency when salty taste was preferred (Table 5). Since preference for salty taste is related to the intake of salty foods, participants who prefer a salty taste need to receive dietary education and diet management for the prevention of GC.

This study has several strengths. It was conducted based on the ranking of major dish groups that contribute to sodium intake in Koreans, and it confirmed the association between GC and the intake of noodles and dumplings, which has not been reported in other studies. In addition, to avoid information bias, interviews of all subjects were conducted without disclosing the disease status after endoscopic examination. In addition, to avoid misclassification bias, controls recruited from the same hospital during the same period were confirmed to have no gastric problems through gastroscopy. This study also has limitations. In our study, the cases were recruited from only two hospitals, the controls were also recruited from the patients who visited the hospital, and they may be less representative of the general population. In addition, the sample for the analysis was small because some cases and controls could not be matched by age, sex, or hospital. There was also a slight difference in the content of the FFQ survey for the first and second stages, and there may have been unintentional misclassification. In addition, we adjusted for various confounding factors in the statistical model; however, there may have been residual confounding effects. Moreover, in our study, the *H. pylori* infection rate was found to be lower in cases than in the controls. This might be due to the fact that *H. pylori* detection rates can decrease with the progression of gastric atrophy and intestinal metaplasia [49,50]. Regarding family history of GC, we previously identified a significant increase in the incidence of GC in subjects with a family history of GC in our previous case-control study (316 pairs) (OR: 1.69, 95% CI: 1.04–2.74) [51]. However, in this study, as the number of subjects was rematched according to the study purpose and reduced to 307 pairs, the significance between the incidence of GC in subjects with a family history of GC disappeared. This is probably due to the fact that the sample size was too small detect the minor contributors to GC.

## 5. Conclusions

Our study showed that the risk of GC increased with the high intake of noodles and dumplings among Koreans, especially in participants who were past or current drinkers. Therefore, in order to prevent and reduce GC, we suggest that Koreans need to reduce their broth intake when eating noodles, make practical efforts to consume as little salt as possible when eating food, and reduce the salt content of noodles themselves as a policy.

## Figures and Tables

**Table 1:** Participant characteristics of gastric cancer cases and controls.

Characteristics	Cases (*n* = 307)	Controls (*n* = 307)	*p*-Values
Age (year, mean ± SD)	56.9 ± 12.0	56.2 ± 11.9	0.450
Sex (*n*, %)			
Men	206 (67.1)	206 (67.1)	1.000
Women	101 (32.9)	101 (32.9)	
Hospital (*n*, %)			
Chungnam University Hospital	124 (40.4)	124 (40.4)	1.000
Hanyang University Guri Hospital	183 (59.6)	183 (59.6)	
Education (*n*, %)			
≤Elementary school	90 (29.3)	92 (30.0)	0.806
Middle school	52 (16.9)	49 (16.0)	
≥High school	136 (44.3)	143 (46.6)	
Missing	29 (9.5)	23 (7.5)	
Family history of gastric cancer (*n*, %) ^†^			
No	244 (79.5)	259 (84.4)	0.146
Yes	46 (15.0)	30 (9.8)	
Missing	17 (5.5)	18 (5.9)	
Body mass index (kg/m^2^) (*n*, %)			
Underweight (≤18.49)	20 (6.5)	13 (4.2)	0.014
Normal weight (18.5–22.99)	134 (43.7)	102 (33.2)	
Overweight (23.0–24.99)	61 (19.9)	69 (22.5)	
Obesity (≥25)	68 (22.2)	100 (32.6)	
Missing	24 (7.8)	23 (7.5)	
*H. pylori* infection (*n*, %)			
No	108 (35.2)	67 (21.8)	<0.001
Yes	97 (31.6)	140 (45.6)	
Missing	102 (33.2)	100 (32.6)	
Total energy intake (kcal/day, mean ± SD)	1870.8 ± 741.5	1700.9 ± 619.6	0.002
Alcohol consumption (*n*, %)			
Never	104 (33.9)	103 (33.6)	0.198
Former	54 (17.6)	39 (12.7)	
Current	149 (48.5)	165 (53.8)	
Smoking status (*n*, %)			
Never	108 (35.2)	126 (41.0)	0.343
Former	93 (30.3)	89 (29.0)	
Current	105 (34.2)	92 (30.0)	
Missing	1 (0.3)	0 (0)	

^†^ first-degree relative.

**Table 2:** Odds ratios of gastric cancer by alcohol consumption or smoking status.

Variables	No. of Cases ^§^/Controls	Model I ^(1)^		Model II ^(2)^	
		OR	(95 % CI)	OR	(95% CI)
Alcohol consumption					
Never	104/103	1.00	Ref.	1.00	Ref.
Ever	203/204	1.00	(0.67–1.49)	2.09	(0.76–5.73)
Smoking status					
Never	108/126	1.00	Ref.	1.00	Ref.
Ever	198/181	1.73	(1.06–2.84) *	1.51	(0.62–3.66)

* *p* < 0.05 compared with reference group. OR, odds ratios; CIs, confidence intervals. ^(1)^ Model I: adjusted for age and sex. ^(2)^ Model II: model I + further adjusted for body mass index (≤18.49, 18.5–22.99, 23.0–24.99, or ≥25), education level (≤elementary school, middle school, ≥high school, or missing), family history of gastric cancer (no, yes, or missing), smoking status (never, past, or current smokers), alcohol drinkers (non, past, or current drinkers), total energy intake (continuous), and *H. pylori* infection (no, yes, or missing). ^§^ There was a missing value in smoking status (cases, *n* = 1).

**Table 3:** Odds ratios of gastric cancer by high-sodium dish groups.

Variables	No. of Cases/Controls	Model I ^(1)^		Model II ^(2)^	
		OR	(95% CI)	OR	(95% CI)
Noodles and dumplings					
Tertile 1	86/120	1.00	Ref.	1.00	Ref.
Tertile 2	106/97	1.58	(1.06–2.34) *	1.31	(0.86–1.99)
Tertile 3	115/90	1.98	(1.31–3.00) **	1.65	(1.03–2.63) *
*p* for trend		<0.001		0.017	
Soups					
Tertile 1	98/106	1.00	Ref.	1.00	Ref.
Tertile 2	101/104	1.09	(0.73–1.62)	1.05	(0.69–1.61)
Tertile 3	108/97	1.26	(0.84–1.90)	1.17	(0.75–1.82)
*p* for trend		0.559		0.927	
Stews					
Tertile 1	105/92	1.00	Ref.	1.00	Ref.
Tertile 2	103/110	0.82	(0.56–1.21)	0.79	(0.52–1.20)
Tertile 3	99/105	0.83	(0.56–1.23)	0.78	(0.51–1.19)
*p* for trend		0.261		0.170	
Pickled vegetables					
Tertile 1	108/96	1.00	Ref.	1.00	Ref.
Tertile 2	96/109	0.78	(0.53–1.15)	0.67	(0.44–1.02) ^†^
Tertile 3	103/102	0.89	(0.60–1.32)	0.80	(0.52–1.24)
*p* for trend		0.510		0.288	

* *p* < 0.05, ** *p* < 0.01, ^†^ *p* < 0.1 compared with reference group. OR, odds ratios; CIs, confidence intervals. ^(1)^ Model I: adjusted for age and sex. ^(2)^ Model II: model I + further adjusted for body mass index (≤18.49, 18.5–22.99, 23.0–24.99, or ≥25), education level (≤elementary school, middle school, ≥high school, or missing), family history of gastric cancer (no, yes, or missing), smoking status (never, past, or current smokers), alcohol drinkers (non, past, or current drinkers), total energy intake (continuous), and *H. pylori* infection (no, yes, or missing).

**Table 4:** Association gastric cancer and intakes of noodles and dumplings according to alcohol consumption or smoking status.

Noodles and Dumplings	No. of Cases/Controls	OR(95%CI) ^‡^	
Alcohol consumption			
Never			
Tertile 1	33/34	1.00	Ref.
Tertile 2	40/32	0.95	(0.46–1.99)
Tertile 3	31/37	0.61	(0.28–1.36)
*p* for trend		0.281	
Ever			
Tertile 1	53/82	1.00	Ref.
Tertile 2	70/66	1.39	(0.82–2.35)
Tertile 3	80/56	2.00	(1.12–3.56) *
*p* for trend		0.017	
Smoking status			
Never			
Tertile 1	35/45	1.00	Ref.
Tertile 2	35/41	0.97	(0.49–1.92)
Tertile 3	38/40	1.06	(0.50–2.22)
*p* for trend		0.817	
Ever			
Tertile 1	57/68	1.00	Ref.
Tertile 2	67/61	1.21	(0.70–2.08)
Tertile 3	74/52	1.68	(0.92–3.05) ^†^
*p* for trend		0.083	

* *p* < 0.05, ^†^ *p* < 0.1 compared with reference group. OR, odds ratios; CIs, confidence intervals. ^‡^ adjusted for age, sex, body mass index (≤18.49, 18.5–22.99, 23.0–24.99, or ≥25), education level (≤elementary school, middle school, ≥high school, or missing), family history of gastric cancer (no, yes, or missing), smoking status (never, past, or current smokers), alcohol drinkers (non, past, or current drinkers), total energy intake (continuous), and *H. pylori* infection (no, yes, or missing). In stratification analysis, the stratified variable was excluded from the model.

**Table 5:** Odds ratios of gastric cancer according to preference of salty food.

Variables	No. of Cases ^§^/Controls	Model I ^(1)^		Model II ^(2)^	
		OR	95%CI	OR	95%CI
Preference of salty food					
Not favored at all and slightly not favored	87/102	1.08	(0.69–1.70)	1.17	(0.72–1.89)
No opinion	56/71	1.00	Ref.	1.00	Ref.
Slightly favored and strongly favored	163/134	1.54	(1.01–2.33) *	1.51	(0.97–2.36) ^†^
*p* for trend		0.046		0.153	

* *p* < 0.05, ^†^ *p* < 0.1 compared with reference group. OR, odds ratios; CIs, confidence intervals. ^(1)^ Model I: adjusted for age and sex. ^(2)^ Model II: model I+ further adjusted for body mass index (≤18.49, 18.5–22.99, 23.0–24.99, or ≥25), education level (≤elementary school, middle school, ≥ high school, or missing), family history of gastric cancer (no, yes, or missing), smoking status (never, past, or current smokers), alcohol drinkers (non, past, or current drinkers), total energy intake (continuous), *H. pylori* infection (no, yes, or missing). ^§^ There was a missing value in the preference for salty food (cases, *n* = 1).

## Data Availability

The data presented in this study are available on request from the corresponding author. The data are not publicly available because of the privacy of the subjects.

## Supplementary Materials

The following are available online at https://www.mdpi.com/article/10.3390/nu13041365/s1, Table S1: Association gastric cancer and intakes of noodles and dumplings according to family history of gastric cancer or *H. pylori* infection.
