# Supplementary material for: Gastric Cancer and the Daily Intake of the Major Dish Groups Contributing to Sodium Intake: A Case-Control Study in Korea
Source: Nutrients. 2021 Apr 19;13(4):1365. doi: 10.3390/nu13041365 (PMC8072798; doi:10.3390/nu13041365)
Supplement: Supplementary file 1 [file nutrients-13-01365-s001.zip › nutrients-1186112-supplementary.pdf]

Table S1. Association gastric cancer and intakes of noodles and dumplings according to family history of gastric cancer or *H. pylori* infection.

Noodles & dumplings	No. of case/control	OR	95 %CI [‡]
**Family history of gastric cancer**			
**No**			
Tertile 1	69/99	1.00	Ref.
Tertile 2	83/87	1.19	(0.75-1.89)
Tertile 3	92/73	1.61	(0.96-2.71)[†]
p-value for trend		0.065	
**Yes**			
Tertile 1	13/11	1.00	Ref.
Tertile 2	14/12	0.52	(0.11-2.54)
Tertile 3	19/7	4.62	(0.79-27.07)[†]
p-value for trend		0.040	
***H. pylori* infection**			
**No**			
Tertile 1	36/23	1.00	Ref.
Tertile 2	32/26	0.59	(0.26-1.36)
Tertile 3	40/18	1.09	(0.45-2.64)
p-value for trend		0.267	
**Yes**			
Tertile 1	24/53	1.00	Ref.
Tertile 2	37/44	1.74	(0.84-3.63)
Tertile 3	36/43	2.18	(0.98-4.88)[†]
p-value for trend		0.020	

[†] $p < 0.1$ compared with reference group.

[‡]adjusted for age, sex, body mass index (≤ 18.49, 18.5-22.99, 23.0-24.99, or ≥ 25), education level (≤ elementary school, middle school, ≥ high school, or missing), family history of gastric cancer (no, yes, or unknown), smoking status (never, past, or current smokers), alcohol drinkers (non, past, or current drinkers), total energy intake (continuous), *H. pylori* infection (no, yes, or missing).
In stratification analysis, the stratified variable was excluded from the model.
